# Ethnic differences in the time trend of female breast cancer incidence: Singapore, 1968 – 2002

**DOI:** 10.1186/1471-2407-6-261

**Published:** 2006-11-02

**Authors:** Xueling Sim, R Ayesha Ali, Sara Wedren, Denise Li-Meng Goh, Chuen-Seng Tan, Marie Reilly, Per Hall, Kee-Seng Chia

**Affiliations:** 1 Centre for Molecular Epidemiology, National University of Singapore and Genome Institute of Singapore, Singapore; 2 Department of Mathematics and Statistics, University of Guelph, Canada; 3 Department of Medical Epidemiology and Biostatistics, Karolinska Institute, Sweden; 4 Department of Paediatrics, National University of Singapore, Singapore; 5 Centre for Molecular Medicine, Biomedical Research Council, Agency for Science, Technology and Research, Singapore; 6 Department of Community, Occupational and Family Medicine, National University of Singapore, Singapore

## Abstract

**Background:**

From 1968 to 2002, Singapore experienced an almost three-fold increase in breast cancer incidence. This increase appeared to be different across the three main ethnic groups: Chinese, Malays and Indians. This paper used age-period-cohort (APC) modelling, to determine the effects of age at diagnosis, calendar period, and birth cohort on breast cancer incidence for each ethnic group.

**Methods:**

This study included all breast cancer cases (n = 15,269) in the three ethnic groups, reported to the Singapore Cancer Registry from 1968 to 2002 between the ages 25 to 79. Age-specific fertility rates from the Department of Statistics were used to explore the role of fertility.

**Results:**

In the 1970s, Indian women had the highest age-standardized breast cancer but by the mid-1980s the highest rates were seen among the Chinese. Remarkable differences were seen in the age-specific incidence rates by ethnic groups. After age 49, the incidence rates for the Chinese and Malays leveled off whereas it continued to rise in the Indians. While our analyses provided some evidence that an age-drift model described the trend seen in the Indians, age-period-cohort model and age-cohort model had the best fit for the Chinese and Malays aged 25 to 79 respectively. Overall, Chinese and Malay women born in later cohorts were at increased risk of developing breast cancer relative to their counterparts in the earlier cohorts. The three ethnic groups experienced similar changes in their fertility in the 1970s, which likely explained much of the increase in their breast cancer incidence but not the ethnic differences. There was a stronger inverse association between total fertility rate and pre-menopausal breast cancer incidence in the Chinese and Malays than the Indians.

**Conclusion:**

The observed dissimilarity among ethnic groups suggests ethnic differences in exposure or response to certain risk factors. It is likely that longer and subtler differences in childbearing trends and other risk factors may further explain these ethnic differences.

## Background

The rapid rise in female breast cancer incidence in Singapore has been attributed to a birth cohort effect that probably arised from the rapid changes in reproductive and other lifestyle patterns [1]. The current breast cancer incidence rate in Singapore is 55 per 100,000 women per year, increasing by approximately 3 percent per year from 1968. Given that large scale genetic mutations do not occur over two or three generations, this increase in incidence can hardly be attributed to changes in genetic complexity [2,3]. Studies of migrant populations allow comparison of genetically similar populations living in different environments [3]. These changes in risks of migrating populations after living in a new environment suggest that the degree of difference by place and over time could be explained by differences in exposures and behaviours [3,4].

Time trends in breast cancer rates may be attributed to an aging population (age effect), changes occurring at a specific calendar period (period effect) or changes affecting persons born at specific times (cohort effect). Hence an age-period-cohort (APC) model can be used to assess the effects of the three factors on disease rates [5,6].

Breast cancer incidence rates have been reported to be different between Asian and Caucasian populations [3,7,8]. Transitioning from a developing to a developed country usually involves the adoption of a "Westernised" lifestyle which includes a combination of decreased parity, delayed childbearing, diet rich in saturated fats, early menarche and a sedentary life pattern [7-9]. These factors have been associated with increased incidence of breast cancer among other risk factors such as family history and high endogenous estrogen levels [10-13]. In the comparative study between Singapore and Sweden, it has been suggested that the larger cohort effect experienced by Singapore could be explained by the effect of a changing environment, particularly reproductive and lifestyle patterns, during its transition to a first world country.

Singapore is characterised by three main ethnic groups: Chinese, Malays and Indians. These three ethnic groups have remarkable differences in breast cancer incidence in spite of relatively similar changes in reproductive and lifestyle changes. Our primary aim is to explore the trends in the incidence rates of breast cancer in the three ethnic groups through the use of APC modelling and if differential reproductive and lifestyle patterns between the ethnic groups could offer some explanation behind any trends observed.

## Methods

This is a retrospective population-based study performed using data from the Singapore Cancer Registry. The Singapore Cancer Registry was established in 1968 where all medical practitioners and pathology laboratories voluntarily notify the registry of any incident cancers. Staff of the Registry also reviews cancer patient hospital discharges and death certificates against registered cases to ensure completeness of records. The completeness of reporting is high: 96% in the 1970s and close to 100% in the 1990s [14]. In addition, the proportions of breast cancer that went unnoticed until the time of death were very low and there was no difference between the three ethnic groups (Chinese: 0.7%; Malays: 1.6%; Indians: 0.9%).

Data on all breast cancer cases among residents diagnosed between 1968 and 2002, stratified by ethnic group (Chinese, Malays, Indians) were obtained from the 5-yearly reports published by the Singapore Cancer Registry [15]. These data included *i) *the number of breast cancer cases in 5-year intervals for both age (0 – 80+) and year of diagnosis (1968 – 2002), *ii) *the 5-year age-specific rates, and *iii) *the age-standardized rates using world standard population [16]. The annual number of live births for the resident population of Singapore in the period 1957 – 2002, the birth order of the live birth and the mother's ethnicity, age at the time of the birth (in 5-year interval) were also obtained from annual reports of the Registrar of Births and Deaths Singapore [17].

As age at first birth and number of children influence breast cancer incidence, scatterplots of total fertility against cumulative breast cancer incidence rates were used to explore the association of breast cancer with total fertility by birth cohorts. Due to incomplete data, we only present scatterplots for the pre-menopausal women (aged 25 – 49) and five birth cohorts in 2-year interval (1943 – 1944, 1945 – 1946, 1947 – 1948, 1949 – 1950, and 1951 – 1953). The cumulative incidence rates estimate the risk of developing breast cancer for a particular birth cohort of women from age 25 to 49, computed from cancer incidences by single age and year of diagnosis requested from the cancer registry. The total fertility rate (TFR) for a particular birth cohort of women estimates the number of children 1,000 of them would bear. To obtain the TFR by cohort from the annual fertility rates in 5-year age groups, we did a linear interpolation between two midpoints of adjacent age groups to obtain the yearly age-specific fertility rates (i.e. assuming linearity in rates across the two age groups). We also plotted the TFR by period (sum of the age-specific birth rates of women in five-year age groups multiplied by five) that estimates the number of children 1,000 women would bear if the age-specific birth rates of a specified calendar-year reflected their childbearing pattern.

APC modelling was used to fit the Singapore breast cancer data. We have breast cancer data in the period 1968 – 2002 (7 calendar periods by 5-year intervals) but restricted the analysis to women aged 25 – 79 (11 age groups by 5-year intervals) because of too few events in the younger age groups and data quality in the oldest age group. In the modelling procedure, we took a model-building approach [6]. The first model incorporated the age because disease incidence usually has a strong age effect. We then fit the age-drift model adding the drift (regular trend) term in the model to be compared to the first model. The drift is the temporal variation of rates indistinguishable as either period or cohort influences. This age-drift model was then compared to the age-period and age-cohort models separately. Finally the full APC model was considered.

For the full APC model, it is well-known that the linear relationship between age, period and cohort does not allow simultaneous estimation of all three effects unless additional assumptions are imposed [5,6]. Therefore, one of the common approaches is to impose a constraint, for example, setting the first two calendar-year periods as identical [18]. This approach has its limitation as the parameter estimates can vary considerably with different constraints. To circumvent this limitation, we estimate and present the curvature effects for each factor in the full APC model as these estimates are identifiable and invariant [19,20].

Poisson regression with a log-link was used by APC modelling techniques to model the number of breast cancer cases. We adjusted for the total number of person-years for each age-period combination through an offset term [21]. Akaike's information criterion (AIC), and deviance tests based on the distance of the predicted rates from the observed breast cancer rates, were used to assess the goodness-of-fit of the models [5,22]. A low AIC and a high goodness-of-fit (g.o.f) test p-value (p > 0.05) indicate a good fit of the model to the data. Likelihood ratio tests were done on nested models to assess variable significance. Analysis was done on 5 yearly rates to avoid having too sparse data. We performed all analyses in the R 2.3.0 statistical software package.

The incidence rate ratios (IRR) obtained from APC models were subsequently used to describe the effects associated with calendar period and birth cohort [21]. We first performed APC analyses on the breast cancer incidence data for the period 1968 – 2002 and ages 25 – 79. As the aetiology of breast cancer is believed to be different in pre-menopausal and post-menopausal women with a characteristic inflection around the age of menopause [4,23], we further fitted separate models for women younger than 50 years and women at least 50 years of age at the time of diagnosis [24].

## Results

**Figure 1:**
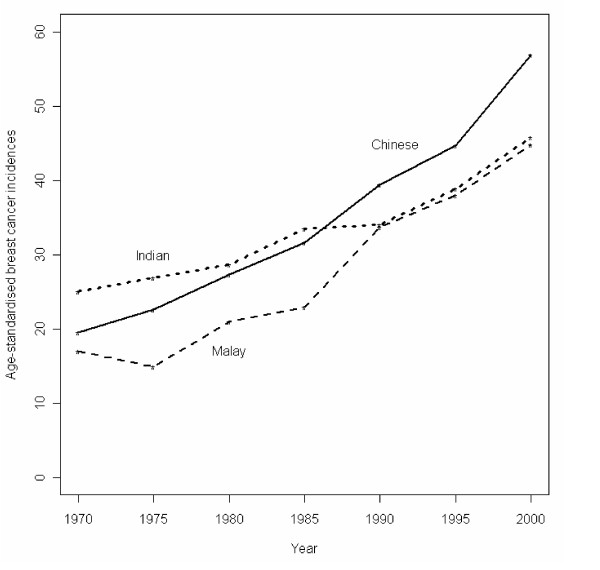
Age-standardized incidence rates of breast cancer stratified by ethnic group from 1968 to 2002 (as indicated by the mid-year of the 5-yearly intervals).

From 1968 to 2002, there were 16,178 breast cancer cases reported to the Singapore Cancer Registry, of which 15,269 were diagnosed in Chinese (85.2%), Malay (9.9%) and Indian (4.9%) women between the ages of 25 to 79. The overall age-standardized incidence rates (Figure 1) were generally increasing over time, with the rates for the Chinese (3.1% per year) and Malays (2.8% per year) increasing at a slightly higher rate than that of the Indians (1.7% per year). The increase in incidence seemed to be more pronounced among Chinese and Malay compared to Indians (Figure 1).

**Figure 2:**
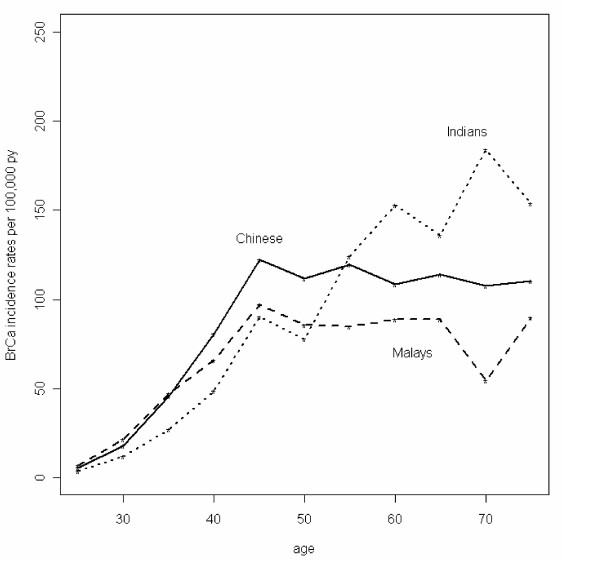
Overall age-specific breast cancer rates stratified by ethnicity and 5-yearly diagnosis period from 1968 – 2002 (as indicated by the first year of the 5-yearly intervals).

The age-specific incidence rates were noticeably different between ethnic groups, particularly so for women after 50 (Figure 2). In women below age 50, breast cancer incidence rates increased with age in all three ethnic groups, with the Indians having the lowest incidence rates. After age 50, the incidence rates for the Chinese and Malays leveled off whereas the postmenopausal rates for Indians continued to rise. For women over age 50, the breast cancer rates were the lowest among Malays and the highest among the Indians (Figure 2).

Using the model building approach [5,6] and ethnicity as an explanatory variable, an age-ethnicity-cohort model with interactions for ethnicity with age and cohort factor provided a good fit to the data (g.o.f p-value = 0.32; AIC = 1419.15). This suggested that age and cohort effects may be different across the ethnic groups, hence separate analyses were performed for each ethnic group.

**Figure 3:**
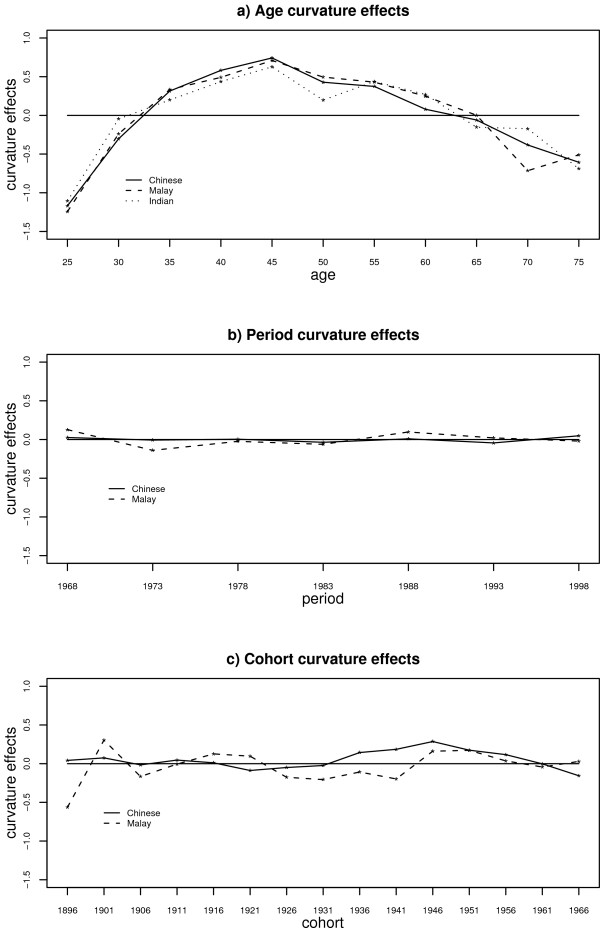
Curvature effects for a) age, b) period and c) cohort from full age-period-cohort model. (First cohort 1891–1895 and last cohort 1971–1975 are not shown as they are only based on one observation).

An age-period-cohort model provided a good fit for Chinese women aged 25 to 79 and an age-cohort model provided reasonable fit for Malays while an age-drift model best fit the Indians (Table 1). Curvature effects, or rate of change effects, were further estimated in the Chinese and Malays. All three ethnic groups had similar age curvature effects (Figure 3a). Period curvature effects (i.e. deviations of the period estimates from the linear periodic trend) were minimal in magnitude (Figure 3b), while large cohort curvature effects suggested departure from linear cohort trends (Figure 3c) for the Chinese and Malays, indicative of dominant cohort curvature effect.

After stratifying the data further into pre- and post-menopausal groups by assuming the median menopausal age to be 50, the age-cohort model provided the best fit for both pre- and post-menopausal Chinese and for pre-menopausal Malays (Table 2). While there was evidence of cohort curvature, it should be noted that there could be underlying linear effect of period in the cohort IRRs; making its interpretation less straightforward. However, for the post-menopausal Malays and the Indians, age-drift models were the best.

Chinese and Malay women born in later birth cohorts had higher risks of developing breast cancer compared to their counterparts born in 1926–1930 (Table 3); but the risk increase was sharper in the pre-menopausal Malays than in the Chinese.

**Figure 4:**
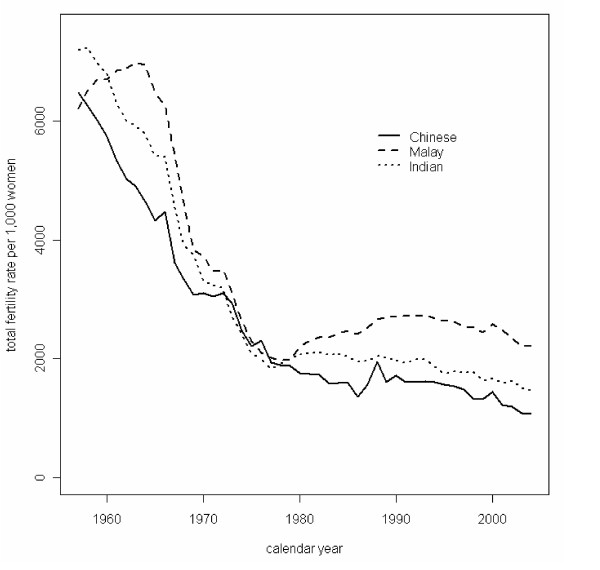
Total fertility rates for Singaporean women aged 15 – 44 from 1968 – 2002, stratified by ethnicity. Source: Reports on Registration of Births and Deaths (1968 – 2002). (Note: Only number of live births in each year is included and female population denominators used are based on interpolation of census data in 1970, 1980, 1990 and 2000).

Total fertility by calendar year declined across the three ethnic groups, from over 4,000 births per 1,000 women in the 1960s to around or less than 2,000 births per 1,000 women in the late 1990s (Figure 4). The Malays tend to have the highest total fertility rate, followed by the Indians and lastly the Chinese. There was also an increasing trend in the age at first birth for the three ethnic groups. The median age at first birth in 1970 ranged from 21.6 to 24.9, while in 2000, it ranged from 25.6 to 29.2 (Table 4). In 1970, the median age of first birth for Malay women was 21.6, compared to 24.9 for the Indians and 23.8 for the Chinese [17]. By the year 2000, the median age had increased to 25.6 for the Malays, 29.2 for the Indians and 28.9 for the Chinese. The Chinese had the highest rate of increase over the 30 years period while the Malays consistently had the median youngest age at first birth.

**Figure 5:**
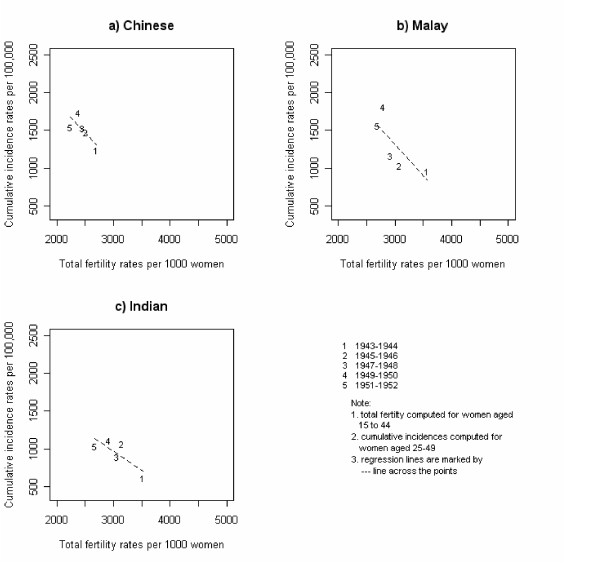
Scatterplots of cumulative breast cancer incidence rates per 100,000 women (for ages 25 to 49) and total fertility per 1,000 women (for ages 15 to 44) for cohorts 1943 – 1944, 1945 – 1946, 1947 – 1948, 1949–1950 and 1951–1952. Over time, decreased total fertility seems to be associated with increased cumulative rate of breast cancer incidence across three ethnic groups.

Scatterplots of cumulative breast cancer incidence rates against total fertility by cohort suggested a difference in the association of total fertility with cumulative incidences between the three ethnic groups (Figure 5). For all three ethnic groups, a decrease in total fertility corresponds to an increase in cumulative incidence rates with the Indians having a weaker magnitude compared to the other two ethnic groups (regression slopes for: Indians -0.51; Chinese -0.81; and Malays -0.83). The more recent cohorts had lower total fertility rates and correspondingly higher cumulative pre-menopausal incidences rates than the earlier cohorts. However, the Chinese had higher cumulative incidence rates and lower total fertility rates compared to the other two ethnic groups for all five 2-year interval birth cohorts.

## Discussion

This study showed that the temporal trends in breast cancer incidence are different among the Chinese, Malays and Indians living in Singapore. It is unlikely that this could be explained by ethnic differences in access to health care as the Ministry of Health in Singapore ensures that universal health coverage is provided to all Singaporeans regardless of their ability to pay [25]. Thus the three ethnic groups have similar access to healthcare but differ with regard to lifestyles, especially in the past. Furthermore, the proportions of breast cancer that went unnoticed until the time of death in the three ethnic groups were very low.

Our data suggest that the temporal trends in Chinese and Malays are most likely attributed to risk factors associated with birth cohort. Specifically, breast cancer incidence in Chinese and Malay women showed a steady increase over birth cohorts from the 1930s, and the age-cohort model provided a good fit for both pre- and post-menopausal Chinese, and pre-menopausal Malays. Results for the Indians indicate that the age-drift models best fit both the pre- and postmenopausal Indians.

The age-specific breast cancer rates between the Indians and the other two ethnic groups also differ. Yansui and Potter suggested that different patterns of age-specific rates may be due to differences in the proportions of ER+ and PR+ breast cancers [26]. Preliminary results on available ER+ and PR+ status of breast cancer tissues did not show any ethnic differences. Based on a series from the largest hospital in Singapore between 1995 and 2005, the proportions of ER+ cases were 65% in Chinese, 62% in Malay and 61% in Indians while those of PR+ cases were 50%, 47% and 50% respectively (unpublished data).

These observed ethnic differences in age-specific rates and results from APC modelling suggest that there may be *i) *ethnic differences in exposure to certain risk factors, or *ii) *ethnic differences in the response to similar changes in exposure, or *iii) *both differences in ethnic exposure and responses.

The changes in breast cancer incidence may be related to Singapore's transition from an industrialized country to a developed country, bringing about changes in lifestyle in the population, including late marriages, delay in childbearing and smaller family sizes. This economic transition led to demographic changes such as low population growth rates, accompanied by changes in socioeconomic status such as increase in incomes and women's participation in paid employment; and higher education levels [27]. Furthermore, in 1966, the Singapore Family Planning and Population Board (SFPPB) introduced family planning campaigns to curb increasing population growth, resulting in a dramatic decline in the total fertility in Singaporean women (Figure 4). However, in the 1980s, pro-natalist family planning programs were introduced in view of the low birth rates and population growth.

From recent data on parity, there was a general decline in number of children born per ever-married female. In 1980, Malay women had the highest mean number of children (3.9) compared to the other two ethnic groups (3.4 for Chinese and for Indians). By year 2000, the numbers declined to 3.1 for the Malays, 2.5 for the Chinese and 2.4 for the Indians [28,29]. The median age at first birth increased in all three ethnic groups from 1970 to 2000 (Table 4). Malay women have consistently had the youngest age at first birth and the highest parity among the three ethnic groups [28-30].

Delayed age at first birth and decreasing parity are possibly important factors governing the changes in breast cancer rates of the Chinese and Malays, and may contribute to the increasing IRRs seen in the birth cohorts after 1930, particularly after 1945. Implementation of the anti-natalist family planning campaign in the early 70s would have had more influence on women in their late teens and twenties, i.e. those who were likely to be born after 1940. While among those who had already given birth we may expect to see lower parity, we would expect to see both delayed first birth and lower parity for the remaining women. This is consistent with the increased rate of change of risk for cohort 1946 – 1950 (Figure 3c) and the marked increase in cohort IRR (Table 3), particularly for the Malay women. Following the changes in family planning after 1980s, the rate of change of risk slowed down in subsequent cohorts from 1951 (Figure 3c).

The impact of delaying first birth and decreasing parity brought by the changes in lifestyle patterns and family planning campaign was probably not as great for the Chinese as it was for the Malays since the Chinese already tended to delay age at first birth and had smaller family sizes relative to the Malays (and Indians) in the 1970s. This is consistent with Figure 3c where the Chinese had an increased rate of change in risk at an earlier cohort (1936–1940) than the Malays (1946–1950). The introduction of family planning campaign may have prolonged the increased rate of change in the Chinese via changes in fertility patterns. These differential changes in age at first birth and parity between the Chinese and Malays may also explain why the increased risk of developing breast cancer observed in Malay women relative to their counterparts in the earlier cohorts is higher than the increased risk of developing breast cancer observed in the Chinese (Table 3).

Although all three ethnic groups experienced delayed age at first birth and decreasing parity, there appears to be ethnic differences in its influence on breast cancer incidence. Decreasing total fertility is found to be associated with increasing cumulative incidence rates in later birth cohorts for Chinese and Malay women aged 25 to 49 (Figure 5). For the Indians, the association was weaker compared to the other two ethnic groups, and the age-drift model fitted them well. The difference may be due to a less pronounced protective effect of reproductive factors in the Indians.

A significant period effect was seen in the Chinese population. While the period curvature effects were minimal compared to the cohort curvature effects, increasing self-awareness of breast cancer in the Chinese could be a contributing factor. The absence of cohort effects in the post-menopausal Malays despite the good age-cohort model fit for the entire Malay population may be due to a cohort influence that is only beginning to emerge in the younger Malay women, particularly those born after 1941 who had just begun to move into their post-menopausal years (≥50).

Our preliminary analyses showed ethnic differences in the association between fertility and pre-menopausal breast cancer. As this cohort matures, the ethnic differences in post-menopausal breast cancer can be further examined. However, total fertility rates may not be an adequate index of the changing fertility patterns as it does not capture the changes in age at first birth of a population, which is known to be a strong protective factor for breast cancer. A record-linkage study between birth registry and cancer registry may confirm the ethnic differences associated with fertility.

Besides reproductive factors, height, dietary habits, obesity, duration of breastfeeding, childhood growth, age at menarche and menopause are some other possible environmental risk factors whose distribution may vary across the ethnic groups, and could also influence breast cancer risk [10,11,31]. Obesity (body mass index ≥ 30 kg/m^2^) has generally been more prevalent among the Malay and Indian women compared to the Chinese women [32-34]. With increasing affluence, there has been an increase in the consumption in all food groups, including dietary fats. From the National Nutrition Survey 2004, the mean fat intake was highest among the Malays (85.2 g), followed closely by the Indians (83.3 g) and lastly the Chinese (76.2 g) [35]. However, it is difficult to establish a direct link between each type of dietary fats and the development of breast cancer since it is difficult to compare trends in consumption of specific food items at ethnic or even population level. Changes in dietary habits, e.g. increased intake of dietary fats, is likely to be confounded with low parity and higher age at first birth which are more common in affluent countries [13]. Thus with possibly limited effect of fertility, the obesity factor may have contributed to an increasing trend in age-specific breast cancer incidence for the Indians. Further, as women progress into sedentary jobs, decreased physical activity could contribute to the cohort effect in the older Chinese women. Breastfeeding has been more common among the Malays [36,37]. As mothers with more children could potentially breastfeed longer, fertility and duration of breastfeeding could be markers of each other. Though there has been an increase in breastfeeding among the better-educated mothers, there is no readily available detailed information about the changes in breastfeeding practices over the study period. The use of menopausal hormones has never been prevalent in any ethnic group in Singapore and are unlikely to explain the observed differences [38].

## Conclusion

This ecological study showed that the temporal trends in breast cancer incidence are different among the Chinese, Malays and Indians in Singapore in spite of fairly similar dramatic declines in fertility rates. Longitudinal cohort data with detailed information on reproductive patterns and other predictive factors will help our understanding of the ethnic differences in the trend of breast cancer incidence in Singapore.

## Competing interests

The author(s) declare that they have no competing interests.

## Authors' contributions

XS, R.AA and KSC conceived of the study, and participated in its design and coordination. XS, R.AA, CST and MR carried out the statistical analysis. SW, DLMG, PH and KSC contributed to the epidemiological aspects. All authors contributed to the writing of the manuscript. All authors read and approved the final manuscript.

## Pre-publication history

The pre-publication history for this paper can be accessed here:



## Figures and Tables

**Table 1:** Model comparison and goodness-of-fit for APC analyses of breast cancer by ethnic groups in Singapore 1968–2002 for ages 25 – 79.

**Population (N)**	Model*	**Deviance**	**df**	**p**	**AIC**
**Chinese Women **(13,003)	**Age**	1534.11	66	<0.001	2058.75
	**Age + drift**	177.74	65	<0.001	704.38
	**Age + period**	168.92	60	<0.001	705.57
	**Age + cohort**	66.76	50	0.06	623.41
	**Age + period + cohort**	**51.92**	**45**	**0.22**	**618.57**

**Malay Women **(1,513)	**Age**	271.88	66	<0.001	620.98
	**Age + drift**	87.03	65	0.04	438.13
	**Age + period**	80.27	60	0.04	441.36
	**Age + cohort**	**59.38**	**50**	**0.17**	**440.48**
	**Age + period + cohort**	53.77	45	0.17	444.86

**Indian Women **(753)	**Age**	78.26	66	0.14	370.08
	**Age + drift**	**54.69**	**65**	**0.82**	**348.52**
	**Age + period**	53.29	60	0.72	357.11
	**Age + cohort**	31.44	50	0.98	355.27
	**Age + period + cohort**	29.30	45	0.97	363.12

*Reference groups are: age group 25 – 29; and period 1968 – 1972 and cohort 1926 – 1930 wherever applicable.

**Table 2:** Goodness-of-fit measures for APC analyses of breast cancer in Singapore, 1968–2002, stratified by age at diagnosis (Age < 50, Age ≥ 50) and ethnicity.

**Population**	Model*	**Age<50**				**Age **≥ **50**			
		
		**Deviance**	**df**	**p**	**AIC**	**Deviance**	**df**	**p**	**AIC**
**Chinese women**	**Age**	641.30	30	<0.001	876.98	892.80	36	<0.001	1181.8
Pre: 6296^†^	**Age + drift**	85.65	29	<0.001	323.32	86.65	35	<0.001	377.62
Post: 6707^‡^	**Age + period**	68.52	24	<0.001	316.19	57.17	30	0.002	358.15
	**Age + cohort**	**29.71**	**20**	**0.07**	**285.38**	**31.07**	**25**	**0.19**	**342.05**
	**Age + period + cohort**	22.61	15	0.09	288.28	22.84	20	0.30	343.82

**Malay women**	**Age**	183.09	30	<0.001	352.17	88.79	36	<0.001	268.80
Pre: 885^†^	**Age + drift**	43.11	29	0.04	214.19	**39.84**	**35**	**0.26**	**221.86**
Post: 628^‡^	**Age + period**	38.05	24	0.03	219.13	37.28	30	0.17	229.29
	**Age + cohort**	**25.98**	**20**	**0.17**	**215.06**	30.46	25	0.21	232.47
	**Age + period + cohort**	22.99	15	0.08	222.07	26.33	20	0.16	238.35

**Indian women**	**Age**	37.64	30	0.16	170.41	40.62	36	0.27	199.67
Pre: 335^†^	**Age + drift**	**30.60**	**29**	**0.38**	**165.38**	**22.93**	**35**	**0.94**	**183.98**
Post: 418^‡^	**Age + period**	29.41	24	0.21	174.18	21.01	30	0.89	192.06
	**Age + cohort**	13.99	20	0.83	166.76	11.80	25	0.99	192.85
	**Age + period + cohort**	13.34	15	0.58	176.12	9.96	20	0.97	201.01

In models for women age below 50, reference groups are: age group 25 – 29; and period 1968 – 1972 and cohort 1926 – 1930 wherever applicable.

In models for women age 50 and above, reference groups are: age group 50 – 54; and period 1968 – 1972 and cohort 1926 – 1930 wherever applicable.

^† ^Number of ethnic specific breast cancer cases among women below age 50.

^‡ ^Number of ethnic specific breast cancer cases among women age 50 and above

**Table 3:** The incidence rate ratio (IRR) for each birth cohort of Chinese and Malay women, relative to the reference cohort (1926 – 1930), estimated from separate models for age < 50 and age ≥ 50 at diagnosis.

	**Age-cohort**			
**Birth Cohort**	Age < 50*		Age ≥ 50*	
	
	**Chinese women**	**Malay women**	**Chinese women**	**Malay women**
	
	**IRR (95% CI)**	**IRR (95% CI)**	**IRR (95% CI)**	**IRR (95% CI)**

**1891–1895**	-	-	0.27 (0.15 – 0.46)	0.60 (0.08 – 4.45)
**1896–1900**	-	-	0.44 (0.33 – 0.57)	0.22 (0.03 – 1.63)
**1901–1905**	-	-	0.53 (0.44 – 0.63)	0.64 (0.29 – 1.41)
**1906–1910**	-	-	0.55 (0.48 – 0.64)	0.47 (0.25 – 0.89)
**1911–1915**	-	-	0.69 (0.61 – 0.77)	0.67 (0.43 – 1.04)
**1916–1920**	-	-	0.77 (0.69 – 0.86)	0.90 (0.64 – 1.27)
**1921–1925**	0.78 (0.59 – 1.04)	1.14 (0.54 – 2.40)	0.83 (0.75 – 0.91)	1.08 (0.80 – 1.45)
**1926–1930**	1.00	1.00	1.00	1.00
**1931–1935**	1.19 (0.98 – 1.44)	1.48 (0.85 – 2.55)	1.21 (1.10 – 1.33)	1.09 (0.81 – 1.45)
**1936–1940**	1.75 (1.47 – 2.09)	1.71 (1.01 – 2.90)	1.62 (1.47 – 1.79)	1.52 (1.13 – 2.04)
**1941–1945**	1.96 (1.65 – 2.32)	1.71 (1.06 – 3.01)	2.10 (1.90 – 2.33)	1.74 (1.25 – 2.42)
**1946–1950**	2.55 (2.17 – 3.00)	3.50 (2.16 – 5.65)	2.82 (2.51 – 3.17)	2.52 (1.76 – 3.60)
**1951–1955**	2.76 (2.35 – 3.24)	4.04 (2.53 – 6.46)	-	-
**1956–1960**	3.09 (2.61 – 3.65)	4.20 (2.58 – 6.83)	-	-
**1961–1965**	3.26 (2.70 – 3.93)	4.60 (2.74 – 7.72)	-	-
**1966–1970**	3.31 (2.60 – 4.21)	5.84 (3.21 – 10.60)	-	-
**1971–1975**	3.03 (1.96 – 4.67)	7.15 (2.85 – 17.93)		

*Empty cells are due to the absence of women diagnosed with breast cancer in the respective birth cohorts.

**Table 4:** Median age at first birth of females in Singapore, stratified by ethnicity and calendar year.

**Ethnic group**	**Calendar year**							**Average rate of increase per year (%)**
		
	**1970**	**1975**	**1980**	**1985**	**1990**	**1995**	**2000**	
**Chinese**	23.77	24.80	25.95	27.08	28.11	28.55	28.93	0.66
**Malay**	21.57	22.25	23.17	23.75	24.81	25.38	25.59	0.57
**Indian**	24.93	25.08	26.16	26.94	28.32	29.07	29.18	0.53
